# Natural Variation of Epstein-Barr Virus Genes, Proteins, and Primary MicroRNA

**DOI:** 10.1128/JVI.00375-17

**Published:** 2017-07-12

**Authors:** Samantha Correia, Anne Palser, Claudio Elgueta Karstegl, Jaap M. Middeldorp, Octavia Ramayanti, Jeffrey I. Cohen, Allan Hildesheim, Maria Dolores Fellner, Joelle Wiels, Robert E. White, Paul Kellam, Paul J. Farrell

**Affiliations:** a Section of Virology, Imperial College Faculty of Medicine, Norfolk Place, London, United Kingdom; b Wellcome Trust Sanger Institute, Hinxton, Cambridge, United Kingdom; c Department of Pathology, VU University Medical Center, Amsterdam, Netherlands; d Laboratory of Infectious Diseases, National Institute of Allergy and Infectious Diseases, Bethesda, Maryland, USA; e Infections and Immunoepidemiology Branch, Division of Cancer Epidemiology and Genetics, NCI, Bethesda, Maryland, USA; f National Institute of Infectious Diseases-ANLIS Carlos G. Malbrán, Buenos Aires, Argentina; g UMR 8126 CNRS, University of Paris-Sud, Institut Gustave Roussy, Villejuif, France; Northwestern University

**Keywords:** BZLF1, EBNA1, Epstein-Barr virus, LMP1, gp350, miRNA

## Abstract

Viral gene sequences from an enlarged set of about 200 Epstein-Barr virus (EBV) strains, including many primary isolates, have been used to investigate variation in key viral genetic regions, particularly LMP1, Zp, gp350, EBNA1, and the BART microRNA (miRNA) cluster 2. Determination of type 1 and type 2 EBV in saliva samples from people from a wide range of geographic and ethnic backgrounds demonstrates a small percentage of healthy white Caucasian British people carrying predominantly type 2 EBV. Linkage of Zp and gp350 variants to type 2 EBV is likely to be due to their genes being adjacent to the EBNA3 locus, which is one of the major determinants of the type 1/type 2 distinction. A novel classification of EBNA1 DNA binding domains, named QCIGP, results from phylogeny analysis of their protein sequences but is not linked to the type 1/type 2 classification. The BART cluster 2 miRNA region is classified into three major variants through single-nucleotide polymorphisms (SNPs) in the primary miRNA outside the mature miRNA sequences. These SNPs can result in altered levels of expression of some miRNAs from the BART variant frequently present in Chinese and Indonesian nasopharyngeal carcinoma (NPC) samples. The EBV genetic variants identified here provide a basis for future, more directed analysis of association of specific EBV variations with EBV biology and EBV-associated diseases.

**IMPORTANCE** Incidence of diseases associated with EBV varies greatly in different parts of the world. Thus, relationships between EBV genome sequence variation and health, disease, geography, and ethnicity of the host may be important for understanding the role of EBV in diseases and for development of an effective EBV vaccine. This paper provides the most comprehensive analysis so far of variation in specific EBV genes relevant to these diseases and proposed EBV vaccines. By focusing on variation in LMP1, Zp, gp350, EBNA1, and the BART miRNA cluster 2, new relationships with the known type 1/type 2 strains are demonstrated, and a novel classification of EBNA1 and the BART miRNAs is proposed.

## INTRODUCTION

Most people in the world are infected by Epstein-Barr virus (EBV) and carry the virus for the rest of their life. EBV infections are usually asymptomatic, but some people develop EBV-associated diseases. Infectious mononucleosis, posttransplant B cell lymphomas, Hodgkin's lymphoma, diffuse large B cell lymphoma, Burkitt's lymphoma (BL), NK-T lymphoma, nasopharyngeal carcinoma (NPC), and gastric carcinoma are diseases associated with EBV (1). Some of those diseases have unusual geographic distribution; in particular, NPC has a high incidence in parts of southern China and in indigenous populations in Malaysia and Indonesia. Thus, relationships between EBV genome sequence variation and health, disease, geography, and ethnicity of the host may be important for understanding the role of EBV in diseases and for development of an effective EBV vaccine.

Recent advances in EBV genome sequencing have resulted in about 130 published EBV genome sequences, the majority of which come from our previous analysis of 84 EBV genomes (2). In that paper, principal-component analysis (PCA) of the unique sequences of the EBV genome showed that the first component of variation is the type 1/type 2 classification, which is determined mainly by EBNA2 and EBNA3. The second component of variation in that data set distinguished Asian strains, particularly those from China and Japan, from the rest of the world.

Type 2 EBV is known to be relatively more frequent in people in sub-Saharan Africa and New Guinea than other parts of the world (3). Several studies have reported type 2 EBV infection in European or U.S. populations but studied either HIV-infected people (4) or healthy subjects and did not define the ethnicity of the EBV-infected people (5). The best-known type 1/type 2 phenotypic difference is the much greater efficiency of B cell immortalization to lymphoblastoid cell lines (LCLs) by type 1 EBV compared to that of type 2 EBV (6). This has been mapped to the EBNA2 locus (7), and amino acid residues that mediate the superior LCL growth maintenance by type 1 EBNA2 have been identified (8). Recent demonstration of T cell infection by type 2 EBV both in cell culture (9) and *in vivo* in Kenyan children (10) has challenged our understanding of the biological significance of type 1 and type 2 EBV and demonstrates the need for more direct studies of this and other aspects of EBV genome variation.

In this paper, we use sections of additional EBV genome sequence that we have determined and our previously published sequence data (2) to focus on variation in specific parts of the EBV genome. This analysis of EBV gene sequences from about 200 EBV strains addresses specific biological questions about EBV genome variation.

## RESULTS

### Healthy white Caucasian British people with type 2 EBV.

**FIG 1:**
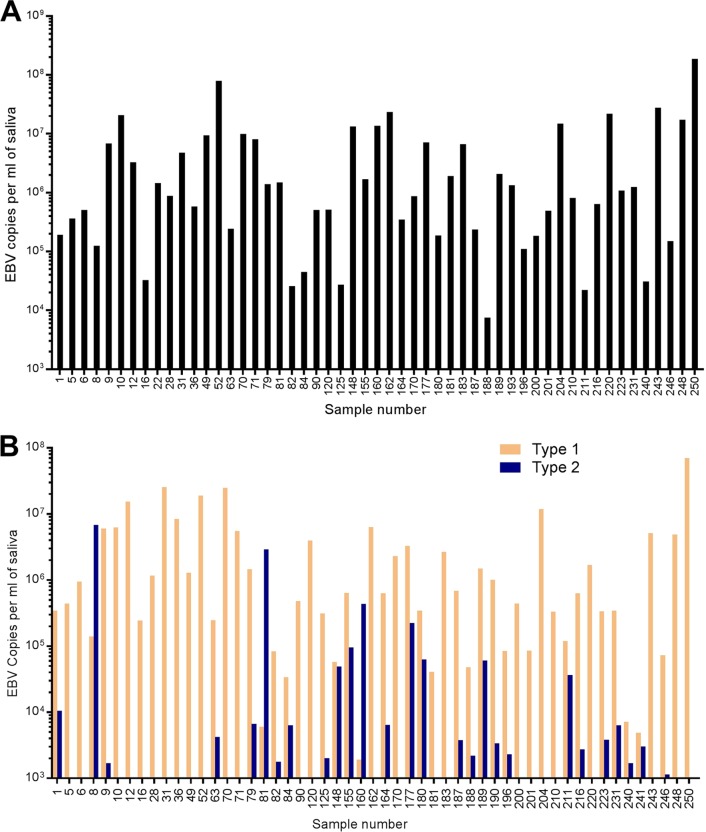
qPCR for BALF5 (A) or EBNA2 (B) type 1 or type 2, corrected for cross talk as described in Materials and Methods. The results are shown for 53 of the 254 saliva samples (see Table S1) that gave more than 10 EBV copies per μl of saliva.

We collected saliva from 254 volunteers who also completed a questionnaire giving information on their clinical history, where they were born and had lived, their ethnic type, and that of their parents (see Table S1 in the supplemental material). DNA was extracted and tested for EBV using PCR; a wide range of viral DNA loads was detected. Fifty-three of the 254 people (20.9%) were found to have 10 EBV genome DNA copies per microliter of saliva or higher by qPCR for EBV using primers in the conserved BALF5 viral DNA polymerase gene (Fig. 1A). Eleven of the 254 participants (4.3%) had a history of infectious mononucleosis (IM), but none reported current IM. A similar fraction (5.6%) of the 53 people who shed more than 10 EBV genome DNA copies per μl saliva had a history of IM. These values were not statistically different (*P* = 0.67 in an unpaired *t* test), so there was no indication that a history of IM is linked to subsequent higher viral shedding in the saliva.

qPCR specific for type 1 or type 2 EBNA2 was then undertaken on the 53 samples that had more than 10 EBV genome DNA copies per μl saliva in the BALF5 test. The results are shown in Fig. 1B. Most of the samples had type 1 EBV, but a significant number of samples had both type 1 and type 2; in a few samples type 2 was predominant. Sample 8 contained 98% type 2 and sample 81 was 99.5% type 2 EBV (Table 1). Donors 8 and 81 (who had no history of IM or any other EBV-related disease) had almost completely type 2 EBNA2, and these people were both white British females with white parents, one from London and the other from Sunderland in the north of England. Sequencing of the type 2-specific PCR products from saliva samples 8, 81, and 160 (and also from 9011, a Taiwanese type 2 saliva EBV; Table S2) showed that these non-African type 2 EBNA2 and EBNA3B sequences were closely related to the reference type 2 AG876 sequence (Fig. S1). We therefore conclude that a small percentage of healthy white British people carry type 2 EBV as their main EBV infection.

**TABLE 1:** Details of 7 saliva donors with type 2 EBV

Sample	EBV type	City of birth	Ethnicity	Gender^*a*^	Age (yr)	Parent ethnicity
8	T2	London, England	White	F	20	White
81	T2	Sunderland, England	White	F	21	White
148	T1, T2	Croydon, England	Asian	F	21	Asian
160	T2	Trivandrum, India	Indian	M	20	Indian
177	T1, T2	Derby, England	Indian	F	20	Indian
189	T1, T2	Rajkot, India	Indian	M	20	Indian
211	T1, T2	Hong Kong, China	Chinese	F	21	Chinese

a F, female; M, male.

### LMP1 classification and EBV type 1 and type 2.

**FIG 2:**
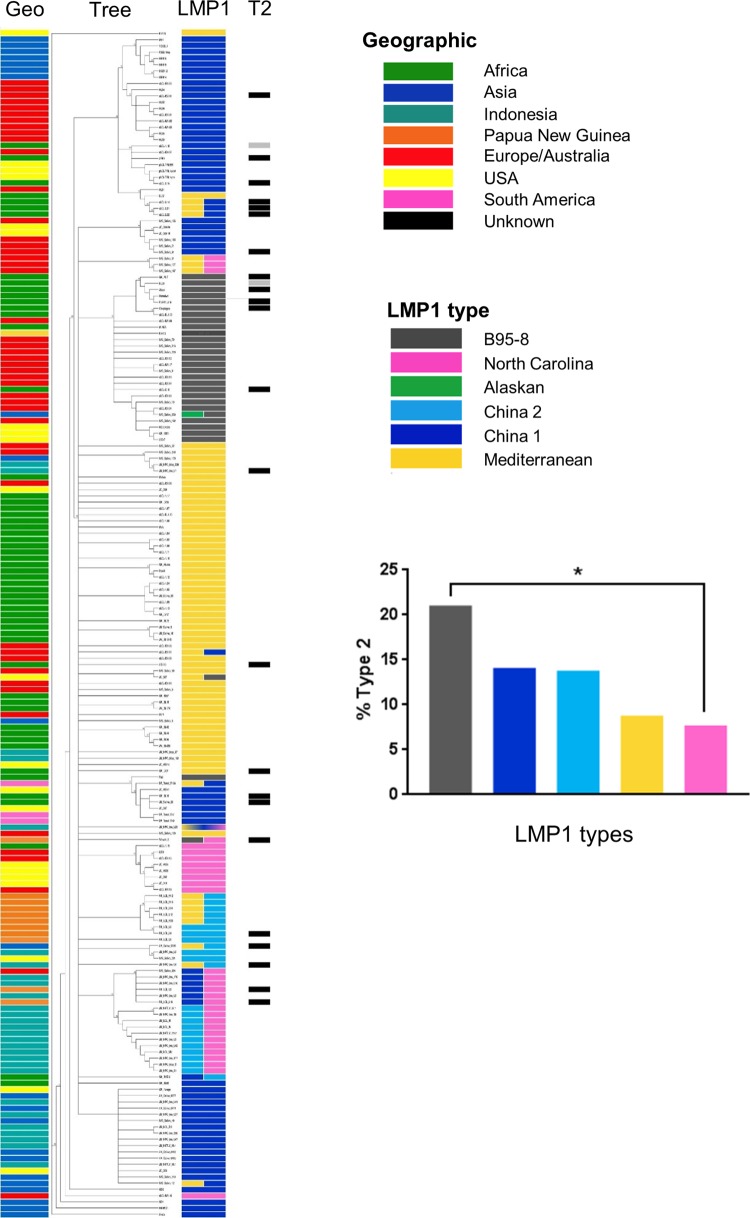
Summary of LMP1 protein sequence phylogenetic tree correlated with LMP1 classification (11), geographic origin (Geo), and type 2 EBV (T2). The percentage of each LMP1 category with type 2 EBV was calculated (samples with mixed classifications were counted as 0.5 for each type). Data including LMP1 protein sequences in a form that can be enlarged to read sample names are shown in Fig. S2. The significant difference in the percentage of type 2 sequences (*P* value of less than 0.02 by one-way analysis of variance) is marked with an asterisk.

Previous genome sequence analysis of EBV (2) indicated that type 1/type 2 is the largest form of natural variation of the EBV genome and was the first component of variation in PCA. The PCA program showed that this variation is mainly determined by EBNA2 and the EBNA3 genes, with a small contribution from LMP1. LMP1 variation has previously been described using a classification based on certain key amino acids (11), and we confirmed this as a simple technique for describing LMP1 variants (2) but did not have sufficient data to explore linkage to type 1/type 2. Using an increased set of 190 LMP1 protein sequences (listed in Fig. S2; details of sources of the sequences are in Table S2), we have now made an enlarged comparison of the classification according to Edwards et al. (11) with the phylogeny of the protein sequences (Fig. 2). There are some intermediate sequences (shown as twin colors in the LMP1 classification), but in general the Edwards et al. LMP1 classification (11) is a reasonable description of the phylogenetic tree, as indicated by the solid blocks of color in the LMP1 column in Fig. 2. However, the geographic origins of the LMP1 alleles are much broader than the names of the LMP1 classification imply (Fig. 2). The EBNA2 type 1/type 2 status of the EBV strains from which the LMP1 protein sequences are derived is also shown in Fig. 2. The B95-8 LMP1 group had the highest association with type 2, and North Carolina had the lowest. These results were significantly different (*P* value of less than 0.02), but the differences in type 2 association between the other groups were not statistically significant.

### Linkage of type1/type 2 classification to haplotypes in Zp promoter and gp350 variation due to their locations in the EBV genome.

Recent studies have identified functionally significant variation in Zp (12) and in the N-terminal part of the gp350 glycoprotein that is required for infection (R. Rochford and N. Smith, personal communication). These forms of EBV genetic variation are partly linked to type 2 EBV, even though those genes were not identified as contributing to type 2 in our principal-component analysis (2).

**FIG 3:**
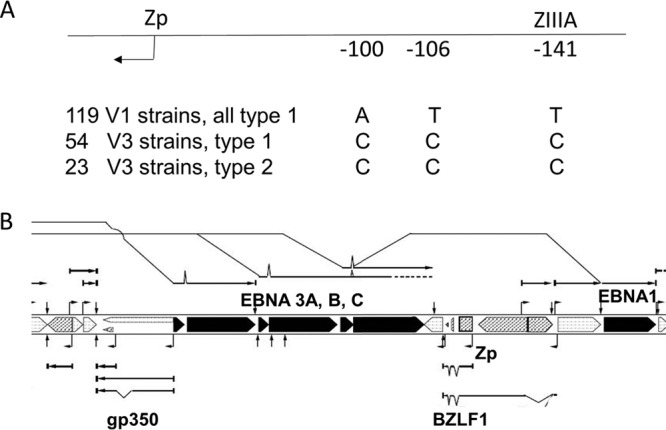
(A) Summary of Zp V1 and V3 haplotypes sorted by C/T at −141 for 196 EBV strains. Full data are shown in Fig. S3. All of the strains with type 2 EBNA genes have the Zp V3 haplotype, but the type 1 strains may have either V1 or V3. (B) Section of EBV genome map showing close proximity of gp350, EBNA3A, EBNA3B, EBNA3C, BZLF1, and Zp but a more distant location of EBNA1 from the EBNA3 locus. The Zp V3 haplotype and gp350 variants are suggested to have originated in type 2 EBV, accounting for the linkage that can be seen in current strains. The map shows the open reading frames (ORFs) of EBV genes, shaded black for latent cycle genes EBNA1, EBNA3A, EBNA3B, and EBNA3C. Structures of mRNAs (some of which are spliced) transcribed rightward (above the ORFs) and leftward (below the ORFs) are also shown.

The results of Bristol et al. (12) showed that the V3 variant of Zp (13) had a greater induction of BZLF1 RNA than the V1 form in a cell-based assay. Comparison of 196 EBV genomes from our enlarged set of EBV genome sequences in the part of Zp containing the three nucleotides that define V3 showed that they sort into two haplotypes (the genome top-strand single-nucleotide polymorphisms [SNPs] are always CCC or ATT) (summarized in Fig. 3A; detailed sequences are shown in Fig. S3). All 23 of the type 2 EBVs had the V3 form at Zp. We suggest that the V3 form of Zp derives originally from type 2 EBV, and the distribution of V1 and V3 that is observed reflects the extensive historic recombination of EBV strains and the proximity of the BZLF1 gene to the EBNA3 locus (Fig. 3B), which contains the most variant genes between type 1 and type 2 EBV.

Although the V3 Zp haplotype can respond more strongly to induction of the EBV lytic cycle in cell culture (12), there was no correlation with EBV shedding by the Zp haplotype in the saliva donors described in Fig. 1. Among 26 high shedders in the 254 saliva donors (Table S1) where Zp was sequenced, the V1 average from 16 saliva samples was 5.7 copies per μl saliva, but the V3 average from 10 saliva samples was 1.9 copies per μl saliva. These values were not significantly different in an unpaired *t* test (*P* = 0.2). Thus, the V3 haplotype does not appear to favor high EBV virus load in the saliva in this set of samples.

Studies of gp350 (BLLF1) variation in published sequences led R. Rochford and N Smith (personal communication) to identify amino acid variation in the N-terminal part of gp350, which was also linked to some extent to type 2 EBV. We have confirmed the linkage of variant amino acids to type 1/type 2 in a larger set of 225 sequences of the N-terminal 120 amino acids of gp350, which contain the residues required for binding to CD21 (Fig. S4). The type 2 EBV strains correspond to a distinct branch of the gp350 phylogenetic tree (Fig. S4). As for the Zp results shown above, the linkage of the gp350 phylogroup to type 2 EBNA3s most likely reflects linkage at the time of the type 1/type 2 divergence and maintenance due to proximity in the genome (Fig. 3B).

The gp350 (amino acids 1 to 120) phylogenetic tree (Fig. S4) shows a large clade of 171 (76% of total) very closely related sequences spanning from Raji to EBVaGC4 which encompass the full range of geographical origins identified and include the reference NC_007605 sequence. However, 24% of the sequences fall into other clades, particularly the type 2 EBV clade and separate clades containing sequences from Indonesia and Papua New Guinea (Fig. S4). It will be important to consider this variation in the part of gp350 to which neutralizing antibodies bind in the design of a universal EBV vaccine.

### EBNA1 DNA binding domain variants.

Protein sequences of EBNA1 were previously classified as P (prototype) or V (variant), each with two subtypes, determined by their amino acid sequence at codon 487 (NC_007605 numbering), giving P-ala, P-thr, V-pro, and V-leu (14). The amino acids used for this classification are located in the DNA binding domain of EBNA1, and it was proposed that some of these EBNA1 subtypes are more likely to lead to oncogenesis, since P-thr and V-leu were found more frequently in samples derived from Burkitt's lymphoma (14).

**FIG 4:**
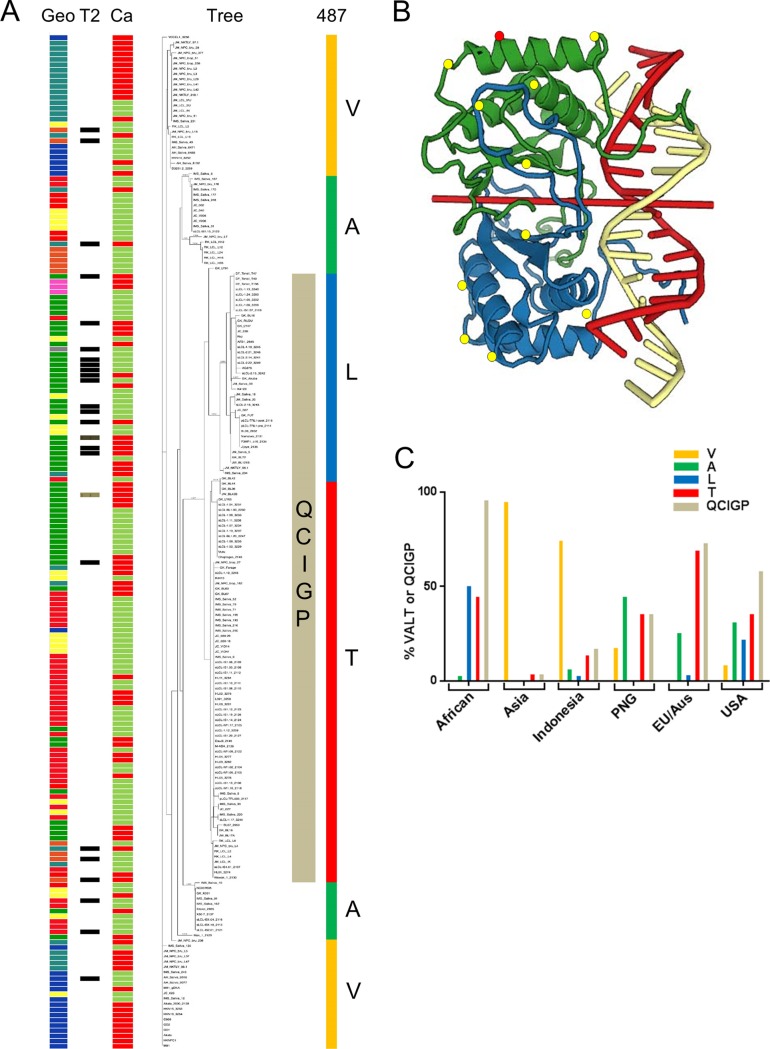
(A) Phylogenetic tree for EBNA1 DNA binding domain (B95-8 EBNA1, amino acids 445 to 614) protein sequence correlated with geographic origin (column Geo; color codes are the same as those in Fig. 2) and EBV type (type 2 strains are marked in the T2 column, and the remainder are type 1). Cancer or normal origin of sample is marked in column Ca (red for cancer, green for normal [see details in Table S2]; spontaneous LCLs from cancer patients are classified as normal in this analysis). The portion of the phylogenetic tree characterized by the QCIGP amino acids is marked, as are the codon 487 amino acids (V, A, L, and T). Sequence data and an enlarged version of the phylogenetic tree in which sample names can be read are shown in Fig. S5. (B) The positions of P476, S492, M563, V574, and T585 of B95-8 EBNA1, which are Q, C, I, G, and P, respectively, in the QCIGP EBNA1 variant are marked as yellow circles on the three-dimensional structure of a dimer of the DNA binding region of B95-8 EBNA1 with its target DNA (entry 1B3T in the RCSB Protein Data Bank). The two subunits of EBNA1 are in green and blue, and the two strands of the DNA are red and yellow. Codon 487 is marked as a red dot on the green subunit (codon 487 on the blue subunit, not visible, is on the back of the structure view shown). (C) Distribution of codon 487 V, A, L, or T and QCIGP in major geographic groups of EBV samples shown in panel A, plotted as a percentage of samples in the indicated geographic group.

To gain a broader understanding of sequence variation in the EBNA1 DNA binding domain (B95-8 EBNA1 amino acids 445 to 614), we have analyzed its sequence variation across the EBNA1 phylogeny by comparing 193 EBNA1 DNA binding domain protein sequences from different parts of the world (Fig. 4A). An expanded version of Fig. 4A, in which the strain names can be read and which also contains the relevant protein sequences, is shown in Fig. S5. The earlier report (14) of EBNA1 variation described codon 487 as A, T, P, or L, but only one example of 487P was reported in that work. It seems that 487P is a very rare variant, because all of the 193 samples we analyzed had A, T, V, or L at codon 487 (Fig. 4A).

The major stratification of these EBNA1 DNA binding domain sequences which can be seen in the tree is marked by the vertical bar in the QCIGP column and is characterized by Q476, C492, I563, G574, and P585 (numbering according to the reference B95-8 NC_007605 EBNA1 protein sequence), in contrast to the sequences P476, S492, M563, V574, and T585 present in B95-8 EBNA1. These amino acids are marked in red in the protein sequences listed in Fig. S5. In the protein structure of the B95-8 EBNA1 DNA binding domain on its DNA binding site (entry 1B3T in the RCSB Protein Data Bank), the amino acids P476, S492, M563, V574, and T585 (marked as yellow dots in Fig. 4B) are not in contact with the bound DNA.

The type 2 EBV strains are indicated in Fig. 4A and Fig. S5. Although there is some clustering of type 2 EBV in the EBNA1 protein tree, this may be mostly related to the geographic origin of the strains. The EBNA1 protein sequences are also identified in Fig. 4A and Fig. S5 as being from cancer or normal cells (saliva or spontaneous LCLs).

The frequency of codon 487 and QCIGP variation differed according to the geographic regions from which the EBV samples were isolated (Fig. 4C). Notably, the African samples were mostly QCIGP and thus 487L or T, whereas the Asian and Indonesian samples were mostly 487V (Fig. 4C). 487T was prominent in the European and Australian samples.

The codon 487A, T, V, and L variants corresponded well to sections of the phylogenetic tree (solid blocks of color in Fig. 4A). Interestingly, codon 487L and T variants corresponded exactly to divisions of the QCIGP group (Fig. 4 and Fig. S5), but there did not appear to be a specific relationship of the L or T variants to BL (14). Most African EBV sequences (53/55, 96%; Fig. S5), and consequently most BL sequences in our study, have L or T at codon 487. Of 26 African BL samples, 13 were 487L (50%) and 12 were 487T (46%). Thus, there does not appear to be a strong association of EBNA1 sequence variation with BL or normal samples beyond the geographic bias within this data set. Codon 487 is marked in Fig. 4B as a red dot on the green subunit (codon 487 on the blue subunit, not visible, is on the back of the structure view shown). It is also not in contact with the bound DNA but might cause changes in the protein structure or affect interaction with other proteins.

### SNPs in the BART cluster 2 region linked to NPC high-incidence area affect BART miRNA expression.

**FIG 5:**
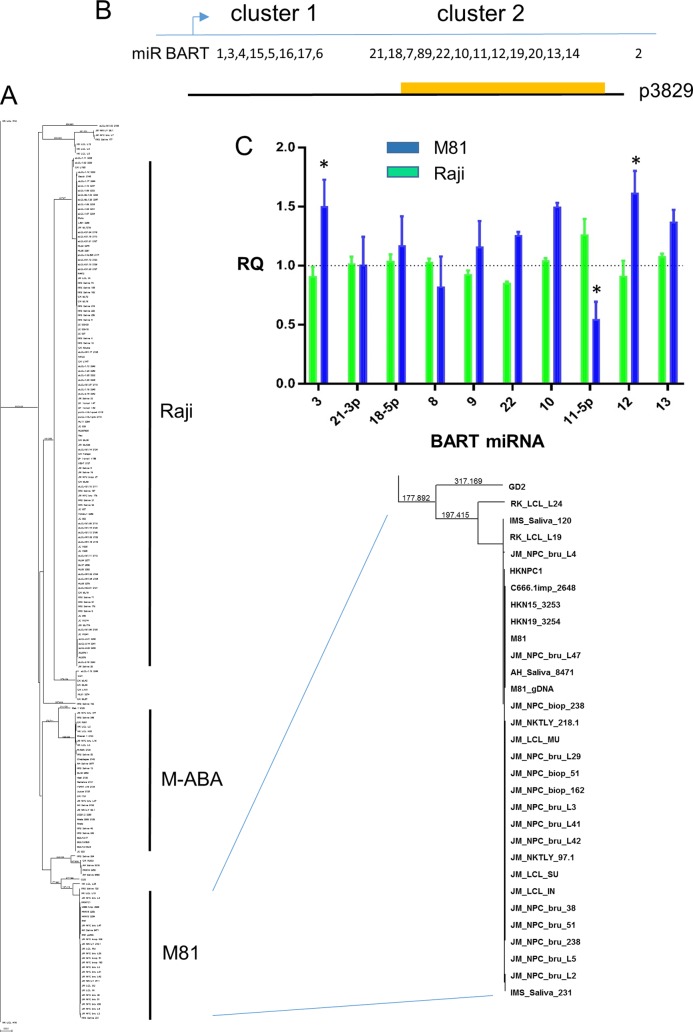
(A) BART cluster 2 phylogenetic tree of EBV DNA sequences with branches characterized by the Raji, M-ABA, and M81 strains marked. An enlarged version of the complete phylogenetic tree and further details are shown in Fig. S6. (B) Schematic of the BART miRNA cluster 1 and cluster 2 EBV genetic regions with relevant miRNA locations marked. The M-ABA EBV plasmid p3829 contains the BART promoter and all of the BART miRNA region except for miR BART2. The SalI fragment containing BART7 to BART14, which was exchanged with Raji or M81 EBV DNA, is shown as a yellow rectangle. (C) BART miRNA expression levels in AGS cell lines expressed as relative quantification (RQ) to the parental M-ABA p3829 plasmid. Results for Raji (green) or M81 (blue) modified p3829 plasmids are shown for BART miRNAs 3, 21-3p, 18-5p, 8, 9, 22, 10, 11-5p, 12, and 13, in the same order as their location in the EBV genome. Data are from RNA from 8 M81, 4 M-ABA, and 2 Raji transfections, each assayed in duplicate, and the means and standard errors are shown relative to the M-ABA value. Values that differed from the M-ABA level with a *P* value of less than 0.002 in a multiple *t* test are marked with an asterisk.

The second principal component (PC2) of our previous analysis of 84 EBV genome sequences (2) separated the Asian EBV strains from the rest of the world. Most of the Asian strains in that data set were from southern China. The high incidence of some EBV-associated cancers in Asian populations, particularly NPC in Cantonese people, suggest a geographic variant that is in some way more oncogenic. The largest contributor to PC2 (2) was LMP1, and variation in LMP1 has been discussed extensively in relation to NPC and tumorigenicity in animal model systems (reviewed in reference 3). The high expression level of BART miRNAs in NPC and demonstration that BART miRNAs can mediate the tumorigenicity of EBV in an animal carcinoma model system (15) is also of interest, because part of PC2 was accounted for by variation in the BART miRNA cluster 2 region (2), which is part of the long pri-miRNA (primary miRNA) transcript from which BART miRNAs are processed. To understand variation in this region of the EBV genome, phylogenetic tree analysis was performed on that part of 195 EBV genomes (Fig. 5A). Accession numbers for the genome sequences supporting this phylogenetic analysis are listed in Table S2. The sequences clustered into 3 main groups and one clade included many Asian NPC genomes (strain names in the phylogenetic tree can be read more easily in Fig. S6, and geographic origins of all samples are listed in Table S2). The sequences of Raji, M-ABA, and M81 were characteristic of the three groups (Fig. 5 and Fig. S6). Comparing the EBV genome sequences of Raji, M-ABA, and M81 in this region showed that only one of the SNPs was in the sequence of a mature BART miRNA (miR BART19), but some of the SNPs are close to miRNA genes, and any of the SNPs could affect the processing of miRNAs.

The BART expression plasmid (p3829) which was used previously to demonstrate the role of BART miRNAs in tumorigenicity (15) contains the M-ABA BART sequence, so we also substituted corresponding Raji or M81 sequences to make three plasmids with BART miRNA cluster 2 regions representative of the three sections of the phylogenetic tree (Fig. 5B). The three BART expression plasmids were transfected into AGS EBV-negative gastric cancer cells, and stably transfected cell lines were selected using hygromycin. The BART miRNA expression levels were determined by TaqMan reverse transcription-PCR (RT-PCR), and the results are presented for some of the more abundant miRNAs as miRNA expression level relative to the M-ABA value (Fig. 5C). The Raji BART region generally gave values similar to those for M-ABA, but some of the M81 miRNAs had significantly different expression levels, for example, miR BART11-5p was lower in M81 and miR BART12 was higher (Fig. 5C). Interestingly, miR BART3 is in cluster 1, which was not changed in the Raji and M81 plasmids (Fig. 5B), but its expression level was affected by the SNPs in the M81 cluster 2 region.

## DISCUSSION

The increasing number of EBV genome sequences available will make it possible to understand more about the natural history of infection, transmission, and relationships between EBV sequence variation and disease. It is noticeable in the phylogeny analyses shown in Fig. 2 to 5 and Fig. S2 to S6 in the supplemental material that the saliva sample EBV sequences we have analyzed are distributed throughout the phylogenetic trees, indicating that the sequences determined from cell lines and clinical samples are not systematically distinct from the virus found in primary isolates, thus helping to validate the whole sequence data set. The predominant variation in EBV appears to be the type 1/type 2 classification, which is primarily determined by EBNA2 and EBNA3 genes. We have shown in this paper that there is some linkage of LMP1 variation to type1/type 2 (consistent with the published principal-component analysis [2]), but geographic bias in type 2 samples in our data set leaves open the possibility that this linkage of LMP1 also is a geographic factor.

Although type 2 EBV is known to have higher incidence in sub-Saharan Africa and Papua New Guinea, previous reports showed the presence of type 2 EBV in western populations at a low level (16), with a higher frequency of type 2 in immunocompromised people (4, 17). However, those studies did not identify the ethnicity of the people infected with type 2 EBV or fully quantify the coinfection with type 1. We have now shown that coinfections are present (Fig. 1B). Selecting the samples with higher EBV load (53/254, 20.8%), we found (Fig. 1B) that about half of these samples had evidence of some degree of coinfection, and a small percentage of white Caucasian people carry type 2 EBV almost exclusively. Because the donors in our study were anonymous, we could not investigate them further. However, it would be interesting to study the cell types infected by EBV in such people, bearing in mind the reported ability of type 2 EBV to infect T lymphocytes (9). The current emphasis on the lesser ability of type 2 EBV to transform B lymphocytes compared to type 1 EBV (6) may miss other important aspects of the biology of type 2 EBV.

The extensive historical recombination between strains discussed in our previous Bootscan analysis (2) is reflected in the linkage of gp350 and Zp variants to type 2 EBV, which can be explained by the proximity of their genes to the EBNA3 locus. The EBNA2 and EBNA3 loci differ so greatly between type 1 and type 2 that they are very unlikely to be able to recombine (18), thereby maintaining these haplotypes in the distinct EBV lineages. A gradient of recombination would be expected in the adjacent genome sequences, and we suggest that this is what is being observed in the gp350 and Zp variants because they are so close to the EBNA3 locus. The very close association of the gp350 and Zp variation with type 2 EBV contrasts with the variation in EBNA1 and LMP1 discussed in this paper; EBNA1 and LMP1 genes are distant in the EBV genome from the EBNA2 and EBNA3 genes, and there was much less association of EBNA1 and LMP1 variation with type 1/type 2. Our previous PCA (2) appeared to restrict the EBNA3 region type 1/2 contribution rather precisely to the EBNA3 genes, with little signal in the adjacent genes. This probably reflects the 1,000-nucleotide window size used in the PCA and very large sequence differences in the EBNA3 gene relative to adjacent genes; it indicates that the PCA is not sufficiently sensitive to detect small differences which are nevertheless biologically significant. It also should be noted that current implementations of the PCA technique only consider SNPs in nucleotides which are present in all sequences being compared; insertions and deletions are routinely excluded from the analysis, so this method may overlook some important forms of variation.

The DNA binding domain of EBNA1 appears to vary in two major forms, which are defined by five amino acids being either PSMVT in the reference strain NC_007605 or QCIGP. The previously described variants (14) reflect this to some degree, as the codon 487L and 487T isolates were exclusively in the QCIGP group (Fig. 4A). Although the QCIGP residues and codon 487 are not thought to contact the DNA directly when bound to the *oriP* EBNA1 binding site, they might affect the overall structure of the domain and the association of other important factors with EBNA1, for example, in the OriP ORC complex or EBNA1 antibody interactions (19). A recent comparison of properties of EBNA1 from M81 EBV (PSMVT, codon 487V) with the reference B95-8 EBNA1 (PSMVT, codon 487A) (20) showed effects on the binding of survivin and a reduced capacity to stimulate DNA replication. Although specific EBNA1 sequence differences have been proposed to contribute to the carcinoma risk of the M81 EBV strain (20), inspection of Fig. S5 shows that none of the amino acid changes discussed (20) is unique to cancer strains. It will be interesting to test effects of the major QCIGP variation on the functional properties of EBNA1 and its interaction with the codon 487 variation.

The different properties of EBV isolated from carcinomas, particularly M81 EBV from an NPC patient, have been proposed to be consistent with EBV in this region being more likely to cause NPC (20–22). Sequence differences between Asian and western EBV strains have been known for some time (2, 23), and our previous principal-component analysis (2) pointed to several regions of the EBV genome that distinguish Asian EBV strains from other EBVs. Focusing on the EBV genome regions which differ in the Asian strains and are strongly expressed in NPC led us to consider LMP1 and BART miRNA cluster 2. LMP1 from NPC EBV has long been considered to be more tumorigenic (24, 25), and here we also find that SNPs that distinguish the M81 BART region cluster 2 can result in expression of different levels of some BART miRNAs. Although the individual differences in expression are only about 1.5- to 2-fold, their cumulative effect might be greater. The mechanism of the different level of miRNA expression from the plasmid with the M81 sequence inserted might involve local SNP effects on processing or complex interactions in the long BART pri-miRNA that could alter the processing efficiency of the miRNAs. Since high levels of BART miRNA expression can mediate the ability of EBV to promote carcinoma development *in vivo* (15), EBV BART region sequence variation could be a factor in the high incidence of EBV-associated carcinoma in southern China and Malaysia.

## MATERIALS AND METHODS

### Saliva samples for EBV PCR analysis.

Saliva was collected from 254 undergraduate student volunteers at Imperial College London by dribbling 5 ml into a 15-ml plastic centrifuge tube, and the samples were then frozen until further use. The samples were collected anonymously, with ethical approval (UK 13/NS/022), in May 2013, and there was a wide range of countries of origin and ethnic types among the participants (see Table S1 in the supplemental material). Additional saliva samples (for phylogeny analyses) were obtained from 12 patients at the National Institutes of Health who signed consent for a protocol that was approved by the Internal Research Board of the National Institute of Allergy and Infectious Diseases. A further 9 saliva samples were from other sources; all of these are listed in Table S2.

### DNA extraction and PCR.

Unless otherwise specified, DNA was isolated as described previously (2). DNA (Table S2) from Indonesian NPC brushings and biopsy specimens (26), Indonesian NK-T lymphoma biopsy specimens (27), Indonesian spontaneous LCLs (28), and Ugandan Burkitt's lymphoma and control samples (29) were purified using silica bead extraction (26).

For the Imperial College student saliva samples (IMS), DNA was extracted from a small sample of each saliva sample using phenol-chloroform, and this was tested (data not shown) for EBV using PCR primers in the BamHI W region (primers GCTAGGCCACCTTCTCAG and GTCCAGGGCCTTCACTTC in GoTaq Flexi buffer, with 30 cycles of 95°C for 30 s, 55°C for 30 s, and 72°C for 30 s). A glyceraldehyde-3-phosphate dehydrogenase (GAPDH) PCR was also performed as a positive control in all of these samples, using primers ATGCCATCACTGCCACCCAGand CCTGCTTCACCACCTTCTTG under the same conditions. A further 2 ml from 53 saliva samples with the highest EBV signals then was extracted with the Oragene OG-500 kit; that DNA was used for experiments shown in Fig. 1A and B and for DNA sequencing where required. TaqMan quantitative PCR (qPCR) was performed for EBV BALF5 with primers CTTTGGCGCGGATCCTC and AGTCCTTCTTGGCTAGTCTGTTGAC with CATCAAGAAGCTGCTGGCGGCC as the probe, normalizing copy numbers to a Namalwa DNA control. Type-specific qPCR for EBNA2 used either a type 1 (TTGTGACAGAGGTGACAAAA) or type 2 (TGGAAGAGTATGTCCTAAGG) forward primer with a common reverse primer (AGGGATGCCTGGACACAAGA) and SYBR green detection under the following PCR conditions: 50°C for 2 min, followed by 95°C for 10 min and then 40 cycles of 95°C for 15 s and 60°C for 1 min. The run method was ended with a melt curve, which was 95°C for 15 s, 60°C for 1 min, and 95°C for 15 s. EBV copy number was normalized to plasmid controls containing type 1 or type 2 EBNA2, and specificity of the type-specific EBNA2 PCR (cross talk) was determined by titrating small amounts of type 1 EBNA2 DNA into the type 2 PCR or vice versa. The cross talk in the assay was less than 0.2%, and the results shown in Fig. 1B have been corrected for this by subtracting 0.2% of the type 1 value from each type 2 result and 0.2% of the type 1 value from each type 2 result.

### DNA sequencing and analysis.

EBV DNA was enriched using the SureSelect method and sequenced as described previously (2). A combination of published *de novo* sequence assemblies (2) and templated sequence assembly for the new genomes was used to derive the sequences shown in this paper. The Wewak EBV sequence, reported previously (2) as Wewak-1, is now considered to be from the Wewak-2 cell line since it is a type 2 EBV. The annotation of the Wewak GenBank file with accession number LN827544 and relevant ENA files are being revised in line with this. Phylogenetic trees of DNA or protein sequences were constructed using the neighbor-joining method with either 1,000 bootstraps or the best tree option of Macvector, version 15.1.4; details are given in the relevant supplemental figures (Fig. S2 to S6).

### BART miRNA analysis.

The plasmid p3829, which contains the M-ABA EBV BART region in a vector (p220) containing *oriP*, EBNA1, and the hygromycin resistance gene, was kindly provided by Bill Sugden. The M-ABA sequence in p3829 is 137581 to 152313 of the M-ABA EBV sequence, accession number LN827527 (from an MluI to a BsaBI restriction site), and includes the BART promoter and all of the BART miRNAs except for BART2. Modifications to p3829 were made by replacing a SalI fragment (146037 to 150336 in EBV reference sequence NC_007605 numbering, which includes miR BART7 to BART14) with equivalent sequence from Raji or M81 EBV (all these strains have the homologous SalI sites in their genome sequences). The DNA sequences of the plasmids throughout the BART region were reconfirmed to ensure that the efficiency of the qPCR assays was not affected by alterations in the miRNA sequence. Plasmids were transfected into AGS cells (an EBV-negative gastric cancer cell line) using 4 × 10^5^ cells in a 6-well plate with 1 μg of DNA (Qiagen EndoFree Plasmid MAXI) and 3 μl of GeneJuice (Novagen) in a 100-μl volume. Twenty-four hours later cells were split, and a further 24 h later they were selected with 0.2 mg/ml hygromycin B to obtain transfected cell lines. RNA was extracted with the mirVana miRNA isolation kit (ThermoFisher), and cDNA was produced from 10 ng RNA using the TaqMan microRNA reverse transcription kit (Applied Biosystems). The miRNA expression was determined by RT-qPCR using the following TaqMan primers from ThermoFisher (catalogue numbers): ebv-miR-BART3* (004432_mat), ebv-miR-bart21-3p (6186), ebv-miR-bart18-5p (8081), ebv-miR-bart8 (8211), ebv-miR-bart9 (7435), ebv-miR-bart22 (6609), ebv-miR-BART10 (004421_mat), ebv-miR-bart11-5p (5755), ebv-miR-bart12 (5725), ebv-miR-bart13 (5446), and U6 snRNA (001973). Levels of miRNAs were first normalized relative to U6 RNA in the same sample and then expressed as shown in Fig. 5C relative to the level in AGS cells containing the M-ABA p3829.

### Accession number(s).

Accession numbers for novel sequences shown in Fig. S1 are MF093903 to MF093906 for EBNA2 and MF093907 to MF093909 for EBNA3B. All other accession numbers for this paper are listed in Table S2.

## Supplementary Material

Supplemental material

## References

[1] Rickinson AB, Kieff E 2007 Epstein-Barr virus, p 2680–2700. *In* Knipe DM, Howley PM, Griffin DE, Lamb RA, Martin MA, Roizman B, Straus SE (ed), Fields virology, 5th ed Lippincott Williams & Wilkins, Philadelphia, PA.

[2] Palser AL, Grayson NE, White RE, Corton C, Correia S, Ba Abdullah MM, Watson SJ, Cotten M, Arrand JR, Murray PG, Allday MJ, Rickinson AB, Young LS, Farrell PJ, Kellam P 2015 Genome diversity of Epstein-Barr virus from multiple tumor types and normal infection. J Virol 89:5222–5237. doi:10.1128/JVI.03614-14. 25787276 PMC4442510

[3] Farrell PJ 2015 Epstein-Barr virus strain variation. Curr Top Microbiol Immunol 390:45–69. 26424643 10.1007/978-3-319-22822-8_4

[4] van Baarle D, Hovenkamp E, Dukers NH, Renwick N, Kersten MJ, Goudsmit J, Coutinho RA, Miedema F, van Oers MH 2000 High prevalence of Epstein-Barr virus type 2 among homosexual men is caused by sexual transmission. J Infect Dis 181:2045–2049. doi:10.1086/315521. 10837190

[5] Higgins CD, Swerdlow AJ, Macsween KF, Harrison N, Williams H, McAulay K, Thomas R, Reid S, Conacher M, Britton K, Crawford DH 2007 A study of risk factors for acquisition of Epstein-Barr virus and its subtypes. J Infect Dis 195:474–482. doi:10.1086/510854. 17230406

[6] Rickinson AB, Young LS, Rowe M 1987 Influence of the Epstein-Barr virus nuclear antigen EBNA 2 on the growth phenotype of virus-transformed B cells. J Virol 61:1310–1317. 3033261 10.1128/jvi.61.5.1310-1317.1987 PMC254104

[7] Cohen JI, Wang F, Mannick J, Kieff E 1989 Epstein-Barr virus nuclear protein 2 is a key determinant of lymphocyte transformation. Proc Natl Acad Sci U S A 86:9558–9562. doi:10.1073/pnas.86.23.9558. 2556717 PMC298536

[8] Tzellos S, Correia PB, Karstegl CE, Cancian L, Cano-Flanagan J, McClellan MJ, West MJ, Farrell PJ 2014 A single amino acid in EBNA-2 determines superior B lymphoblastoid cell line growth maintenance by Epstein-Barr virus type 1 EBNA-2. J Virol 88:8743–8753. doi:10.1128/JVI.01000-14. 24850736 PMC4136291

[9] Coleman CB, Wohlford EM, Smith NA, King CA, Ritchie JA, Baresel PC, Kimura H, Rochford R 2015 Epstein-Barr virus type 2 latently infects T cells, inducing an atypical activation characterized by expression of lymphotactic cytokines. J Virol 89:2301–2312. doi:10.1128/JVI.03001-14. 25505080 PMC4338898

[10] Simbiri KO, Smith NA, Otieno R, Wohlford EE, Daud II, Odada SP, Middleton F, Rochford R 2015 Epstein-Barr virus genetic variation in lymphoblastoid cell lines derived from Kenyan pediatric population. PLoS One 10:e0125420. doi:10.1371/journal.pone.0125420. 25933165 PMC4416826

[11] Edwards RH, Seillier-Moiseiwitsch F, Raab-Traub N 1999 Signature amino acid changes in latent membrane protein 1 distinguish Epstein-Barr virus strains. Virology 261:79–95. doi:10.1006/viro.1999.9855. 10441557

[12] Bristol J, Djavadian R, Barlow E, Ohashi M, Johannsen E, Kenney S 2016 Abstr 17th Int Symp EBV Assoc Dis, abstr EB2016-1185.

[13] Gutierrez MI, Ibrahim MM, Dale JK, Greiner TC, Straus SE, Bhatia K 2002 Discrete alterations in the BZLF1 promoter in tumor and non-tumor-associated Epstein-Barr virus. J Natl Cancer Inst 94:1757–1763. doi:10.1093/jnci/94.23.1757. 12464647

[14] Bhatia K, Raj A, Guitierrez MI, Judde JG, Spangler G, Venkatesh H, Magrath IT 1996 Variation in the sequence of Epstein Barr virus nuclear antigen 1 in normal peripheral blood lymphocytes and in Burkitt's lymphomas. Oncogene 13:177–181. 8700544

[15] Qiu J, Smith P, Leahy L, Thorley-Lawson DA 2015 The Epstein-Barr virus encoded BART miRNAs potentiate tumor growth in vivo. PLoS Pathog 11:e1004561. doi:10.1371/journal.ppat.1004561. 25590614 PMC4295875

[16] Crawford DH, Macsween KF, Higgins CD, Thomas R, McAulay K, Williams H, Harrison N, Reid S, Conacher M, Douglas J, Swerdlow AJ 2006 A cohort study among university students: identification of risk factors for Epstein-Barr virus seroconversion and infectious mononucleosis. Clin Infect Dis 43:276–282. doi:10.1086/505400. 16804839

[17] Yao QY, Croom-Carter DS, Tierney RJ, Habeshaw G, Wilde JT, Hill FG, Conlon C, Rickinson AB 1998 Epidemiology of infection with Epstein-Barr virus types 1 and 2: lessons from the study of a T-cell-immunocompromised hemophilic cohort. J Virol 72:4352–4363. 9557725 10.1128/jvi.72.5.4352-4363.1998 PMC109665

[18] McGeoch DJ, Gatherer D 2007 Lineage structures in the genome sequences of three Epstein-Barr virus strains. Virology 359:1–5. doi:10.1016/j.virol.2006.10.009. 17097710

[19] Middeldorp JM 2015 Epstein-Barr virus-specific humoral immune responses in health and disease. Curr Top Microbiol Immunol 391:289–323. 26428379 10.1007/978-3-319-22834-1_10

[20] Dheekollu J, Malecka K, Wiedmer A, Delecluse HJ, Chiang AK, Altieri DC, Messick TE, Lieberman PM 2017 Carcinoma-risk variant of EBNA1 deregulates Epstein-Barr virus episomal latency. Oncotarget 8:7248–7264. doi:10.18632/oncotarget.14540. 28077791 PMC5352318

[21] Tsai MH, Lin X, Shumilov A, Bernhardt K, Feederle R, Poirey R, Kopp-Schneider A, Pereira B, Almeida R, Delecluse HJ 2017 The biological properties of different Epstein-Barr virus strains explain their association with various types of cancers. Oncotarget 8:10238–10254. doi:10.18632/oncotarget.14380. 28052012 PMC5354655

[22] Tsai MH, Raykova A, Klinke O, Bernhardt K, Gartner K, Leung CS, Geletneky K, Sertel S, Munz C, Feederle R, Delecluse HJ 2013 Spontaneous lytic replication and epitheliotropism define an Epstein-Barr virus strain found in carcinomas. Cell Rep 5:458–470. doi:10.1016/j.celrep.2013.09.012. 24120866

[23] Zhou L, Chen JN, Qiu XM, Pan YH, Zhang ZG, Shao CK 2017 Comparative analysis of 22 Epstein-Barr virus genomes from diseased and healthy individuals. J Gen Virol 98:96–107. doi:10.1099/jgv.0.000699. 28036243

[24] Hu LF, Zabarovsky ER, Chen F, Cao SL, Ernberg I, Klein G, Winberg G 1991 Isolation and sequencing of the Epstein-Barr virus BNLF-1 gene (LMP1) from a Chinese nasopharyngeal carcinoma. J Gen Virol 72(Part 10):2399–2409. doi:10.1099/0022-1317-72-10-2399. 1681026

[25] Hu LF, Chen F, Zheng X, Ernberg I, Cao SL, Christensson B, Klein G, Winberg G 1993 Clonability and tumorigenicity of human epithelial cells expressing the EBV encoded membrane protein LMP1. Oncogene 8:1575–1583. 8389032

[26] Ramayanti O, Juwana H, Verkuijlen SA, Adham M, Pegtel MD, Greijer AE, Middeldorp JM 2017 Epstein-Barr virus mRNA profiles and viral DNA methylation status in nasopharyngeal brushings from nasopharyngeal carcinoma patients reflect tumor origin. Int J Cancer 140:149–162. doi:10.1002/ijc.30418. 27600027 PMC5129462

[27] Adham M, Greijer AE, Verkuijlen SA, Juwana H, Fleig S, Rachmadi L, Malik O, Kurniawan AN, Roezin A, Gondhowiardjo S, Atmakusumah D, Stevens SJ, Hermani B, Tan IB, Middeldorp JM 2013 Epstein-Barr virus DNA load in nasopharyngeal brushings and whole blood in nasopharyngeal carcinoma patients before and after treatment. Clin Cancer Res 19:2175–2186. doi:10.1158/1078-0432.CCR-12-2897. 23493345

[28] Hutajulu SH, Hoebe EK, Verkuijlen SA, Fachiroh J, Hariwijanto B, Haryana SM, Stevens SJ, Greijer AE, Middeldorp JM 2010 Conserved mutation of Epstein-Barr virus-encoded BamHI-A Rightward Frame-1 (BARF1) gene in Indonesian nasopharyngeal carcinoma. Infect Agents Cancer 5:16. doi:10.1186/1750-9378-5-16. 20849661 PMC2949665

[29] Orem J, Sandin S, Mbidde E, Mangen FW, Middeldorp J, Weiderpass E 2014 Epstein-Barr virus viral load and serology in childhood non-Hodgkin's lymphoma and chronic inflammatory conditions in Uganda: implications for disease risk and characteristics. J Med Virol 86:1796–1803. doi:10.1002/jmv.23988. 24889739

