# Supplementary material for: Natural Variation of Epstein-Barr Virus Genes, Proteins, and Primary MicroRNA
Source: J Virol. 2017 Jul 12;91(15):e00375-17. doi: 10.1128/JVI.00375-17 (PMC5512239; doi:10.1128/JVI.00375-17)
Supplement: Supplemental material [file supp_91_15_e00375-17__index.html]

Natural Variation of Epstein-Barr Virus Genes, Proteins, and Primary MicroRNA — Supplemental material 

# Natural Variation of Epstein-Barr Virus Genes, Proteins, and Primary MicroRNA

## Supplemental material

- Supplemental file 1 -

  Fig. S1 (Sequence alignments of type 2 EBNA2 protein and part of EBNA3B DNA from saliva samples, with AG876 as the type 2 reference sequence.)

  Fig. S3 (DNA sequences of Zp V3 region summarized in Fig 3A, shown as EBV genome top strand.)

  Fig. S6 (Phylogenetic tree of BART cluster 2 sequences summarized in Fig. 5.)

  PDF, 379K
- Supplemental file 2 -

  Fig. S2 (LMP1 protein sequence phylogeny related to geographic origin of EBV type1/type 2 and LMP1 classification.)

  XLSX, 3.6M
- Supplemental file 3 -

  Fig. S4 (Protein sequences of gp350 analyzed for Fig. 3B.)

  XLSX, 327K
- Supplemental file 4 -

  Fig. S5 (EBNA1 DNA binding domain sequences analyzed in Fig. 4 with additional Fig 4. details.)

  XLSX, 184K
- Supplemental file 5 -

  Table S1 (Details of the saliva donors analyzed in Fig. 1.)

  XLSX, 36K
- Supplemental file 6 -

  Table S2 (List of samples analyzed by DNA sequencing with geographic origin.)

  XLSX, 20K
