# Supplementary material for: Natural Variation of Epstein-Barr Virus Genes, Proteins, and Primary MicroRNA
Source: J Virol. 2017 Jul 12;91(15):e00375-17. doi: 10.1128/JVI.00375-17 (PMC5512239; doi:10.1128/JVI.00375-17)

Figure S1 Sequence alignments of type 2 EBNA2 protein and part of EBNA3B DNA from saliva samples (AG876 is the type 2 reference sequence).

Type 2 EBNA2 and EBNA3B sequences from saliva samples are similar to the reference type2 AG876 sequence. Saliva samples are 9011 (Taiwan), 160 (Indian Male), 81 (English Female), 08 (English Female.

EBNA2 protein sequence deduced from DNA sequence of PCR products

9011 MPTYYLALHGGQSYNLIVDTDMSGNPSLSVIPTNPYQEQLSNNPLIQLQIVVGENTGAPA
160 MPTYYLALHGGQSYNLIVDTDMSGNPSLSVIPTNPYQEQLSNNPLIQLQIVVGENTGAPA
81 MPTYYLALHGGQSYNLIVDTDMSGNPSLSVIPTNPYQEQLSNNPLIQLQIVVGENTGAPA
08 MPTYYLALHGGQSYNLIVDTDMSGNPSLSVIPTNPYQEQLSNNPLIQLQIVVGENTGAPA
AG876 MPTYYLALHGGQSYNLIVDTDMSGNPSLSVIPTNPYQEQLSNNPLIQLQIVVGENTGAPA
****

9011 PPQPPPPPPPPPPPERRDAWTQEPLPLDMNPLGSDASQGPLASSIRMLCMAQYLLRNARG
160 PPQPPPPPPPPPPPERRDAWTQEPLPLDMNPLGSDASQGPLASSIRMLCMAQYLLRNARG
81 PPQPPPPPPPPPPPERRDAWTQEPLPLDMNPLGSDASQGPLASSIRMLCMAQYLLRNARG
08 PPQPPPPPPPPPPPERRDAWTQEPLPLDMNPLGSDASQG**H**LASSIRMLCMAQYLLRNARG
AG876 PPQPPPPPPPPPPPERRDAWTQEPLPLDMNPLGSDASQGPLASSIRMLCMAQYLLRNARG
*************************************** ********************

9011 QQGLLRPLGPQTRSQVTLERQPVHNPRQEAPIILLQSPAPPRFTPVPMVALGHTLQPTPP
160 QQGLLRPLGPQTRSQVTLGRQPVHNPRQEAPIILLQSPEPPRFTPVPMVALGHTRQPTPP
81 QQGLLRPLGPQTRSQVTLERQPVHNPRQEAPIILLQSPAPPRFTPVPMVALGHTLQPTPP
08 QQGLLRPLGPQTRSQVTLERQPVHNPRQEAPIILLQSPAPPRFTPVPMVALGHTLQPTPP
AG876 QQGLLRPLGPQTRSQVTLERQPVHNPRQEAPIILLQSPAPPRFTPVPMVALGHTLQPTPP
****************** ******************* *************** *****

9011 PRPTLPQPRIPLIIPPRHTNQPATTPPTAPQRLTLGHQLSLPPHPPPHQSTPHCSSDSTG
160 PRPTLPQPRIPLIIPPRHTNQPATTPPTAPQRLTLGHQLSLPPHPPPHQSTPHCSSDSTG
81 PRPTLPQPRIPLIIPPRHTNQPATTPPTAPQRLTLGHQLSLPPHPPPHQSTPHCSSDSTG
08 PRPTLPQPRIPLIIPPRHTNQPATTPPTAPQRLTLGHQLSLPPHPPPHQSTPHCSSDSTG
AG876 PRPTLPQPRIPLIIPPRHTNQPATTPPTAPQRLTLGHQLSLPPHPPPHQSTPHCSSDSTG
****

9011 **P**PPPP**I**SYSIPSMTLSPEPLPPPAAPAHPLPGVIYDQQALPPTPGPPWWPPVRDPTPTTQ
160 LPPPPTSYSIPSMTLSPEPLPPPAAPAHPLPGVIYDQQALPPTPGPPWWPPVRDPMPTTQ
81 LPPPPTSYSIPSMTLSPEPLPPPAAPAHPLPGVIYDQQALPPTPGPPWWPPVRDPTPTTQ
08 LPPPPTSYSIPSMTLSPEPLPPPAAPAHPLPGVIYDQQALPPTPGPPWWPPVRDPTPTTQ
AG876 LPPPPTSYSIPSMTLSPEPLPPPAAPAHPLPGVIYDQQALPPTPGPPWWPPVRDPTPTTQ
 **** *** ****

9011 TPPTNTKQGPDQGQGRGRWRGR------GRMHKLPEPRRPGPDTSSPSMPQLSPVVSLHQ
160 TPPTNTKQGPDQGQGRGRWRGRGRSKGRGRMHKLPEPRRPGPDTSSPSMPQLSPVVSLHQ
81 TPPTNTKQG**T**DQGQGRGRWRGRGRSKGRGRMHKLPEPRRPGPDTSSPSMPQLSPVVSLHQ
08 TPPTNTKQGPDQGQGRGRWRGRGRSKGRGRMHKLPEPRRPGPDTSSPSMPQLSPVVSLHQ
AG876 TPPTNTKQGPDQGQGRGRWRGRGRSKGRGRMHKLPEPRRPGPDTSSPSMPQLSPVVSLHQ
********* ************   ********************************

9011 GQGPENSPTPGPSTAGPVCRVTPSATPDISPIHEPESSDSEEPPFLFPSDWYPPTLEPAE
160 GQGPENSPTPGPSTAGPVCRVTPSATPDISPIHEPESSDSEEPPFLFPSDWYPPTLEPAE
81 GQGPENSPTPGPSTAGPVCRVTPSATPDISPIHEPESSDSEEPPFLFPSDWYPPTLEPAE
08 GQGPENSPTPGPSTAGPVCRVTPSATPDISPIHEPESSDSEEPPFLFPSDWYPPTLEPAE
AG876 GQGPENSPTPGPSTAGPVCRVTPSATPDISPIHEPESSDSEEPPFLFPSDWYPPTLEPAE
****

9011 LDESWEGIFETTESHSSDEENVGGPSKRPRTSTQ
160 LDESWEGIFETTESHSSDEENVGGPSKRPRTSTQ
81 LDESWEGIFETTESHSSDEENVGGPSKRPRTSTQ
08 LDESWEGIFETTESHSSDEENVGGPSKRPRTSTQ
AG876 LDESWEGIFETTESHSSDEENVGGPSKRPRTSTQ

```
                   ***********************************
```

EBNA3B DNA sequence from PCR products

```
B95-8_3B         901 ACGAAAAACCGAGTTTCATTTATTGAGTTTGTAGGATGGTTATGCAAGAAGGACCACACT  960
saliva_8_3B          ACGAGAAACCGAGTTGCATTTGTCGAGTTTGTAGGATGGCTATGTAAGAAGGACCACACT
Saliva_81_3B         ACGAGAAACCGAGTTGCATTTGTCGAGTTTGTAGGATGGCTATGTAAGAAGGACCACACT
Saliva_160_3B        ACGAGAAANCGAGTTGCATTTGTTGAGTTTGTAGGATGGCTATGTAAGAAGGACCACACT
AG876_3B             ACGAGAAACCGAGTTGCATTTGTCGAGTTTGTAGGATGGCTATGTAAGAAGGACCACACT
                     **** ********** ***** * *************** **** ***************

B95-8_3B         961 CATATACGCGAATGGTTCCGCCAGTGCACCGGGAGACCCAAAGCAGCCAAGCCATGGTTA 1020
saliva_8_3B          CATATACGTGAATGGTTCCGCCAGTGCACCGGGAGACCCTCACCATCCAAGCCATGGATG
Saliva_81_3B         CATATACGTGAATGGTTCCGCCAGTGCACCGGGAGACCCTCACCATCCAAGCCATGGATG
Saliva_160_3B        CATATACGTGAATGGTTCCGCCAGTGCACCGGGCGACCCTCACCATCCAAGCCATGGATG
AG876_3B             CATATACGTGAATGGTTCCGCCAGTGCACCGGGAGACCCTCACCATCCAAGCCATGGATG
                     ******** ************************ *****   * ** *********** *

B95-8_3B        1021 AGAGCGCATCCTGTCGCCATTCCTTATGATGATCCGTTAACAAACGAGGAGATTGATCTG 1080
saliva_8_3B          AGAGCGCATCCCGTCGCCGTTCCTTATGACGATCCGTTAACAAGTGAGGAGACTGACCTG
Saliva_81_3B         AGAGCGCATCCCGTCGCCGTTCCTTATGACGATCCGTTAACAAGTGAGGAGACTGACCTG
Saliva_160_3B        AGAGCGCATCCCGTCGCCGTTCCTTATGATGATCCGTTAACAAGTGAGGAGACTGACCTG
AG876_3B             AGAGCGCATCCCGTCGCCGTTCCTTATGACGATCCATTAACAAGTGAGGAGACTGACCTG
                     *********** ****** ********** ***** *******  ******* *** ***

B95-8_3B        1081 GCCTATGCCCGCGGGCAGGCCATGAATATTGAGGCTCCTAGACTGCCAGATGATCCT-AT 1139
saliva_8_3B          GCCTATGCCCGTGGACTGGCCATGAGTATCGAGGCTGCTAGACTGCCAGATGATCCA-AT
Saliva_81_3B         GCCTATGCCCGTGGACTGGCCATGAGTATCGAGGCTGCTAGACTGCCAGATGATCCATAT
Saliva_160_3B        GCCTATGCCCGTGGACTGGCCATGAGTATCGAGGCTGCTAGACTGCCAGATGATCCA-AT
AG876_3B             GCCTATGCCCGTGGACTGGCCATGAGTATCGAGGCTGCTAGACTGCCAGATGATCCA-AT
                     *********** ** * ******** *** ****** *******************  **

B95-8_3B_       1140 AATTGTTGAGGATGACGACGAAAGTGAGGAAATTGAAGCTGAAAGCG  1186
saliva_8_3B          AATTGTTGAGGATGACGATGAAAGTGAGGAAATTGAAGATAAATGTG
Saliva_81_3B         AATTGTTGAGGATGACGATGAAAGTGAGGAAAT-GAAGATAAATGTG
Saliva_160_3B        AATTGTTGAGGATGACGATGAAAGTGAGGAAATTGAAGATAAATGTG
AG876_3B             AATTGTTGAGGATGACGATGAAAGTGAGGAAATTGAAGATAAATGTG
                     ****************** ************** **** * ** * *
```

Figure S3 DNA Sequences of Zp V3 region summarized in Fig 3A, shown as EBV genome top strand.

Zp alignment, sorted by C/T at -141

```
                          -100   -106                               -141
ATT                         *      *                                  *
NC007605        1 GGTGTGTCTATGAGGTACATTAGCAATGCCTGTGGCTCATGCATAGTTTCTAAAAGAGG 59
JM_Saliva_5     1 GGTGTGTCTATGAGGTACATTAGCAATGCCTGTGGCTCATGCATAGTTTCTAAAAGAGG 59
JM_NPC_bru_L2   1 GGTGTGTCTATGAGGTACATTAGCAATGCCTGTGGCTCATGCATAGTTTCTAAAAGAGG 59
JM_NPC_bru_L5   1 GGTGTGTCTATGAGGTACATTAGCAATGCCTGTGGCTCATGCATAGTTTCTAAAAGAGG 59
JM_NPC_bru_L16  1 GGTGTGTCTATGAGGTACATTAGCAATGCCTGTGGCTCATGCATAGTTTCTAAAAGAGG 59
JM_NPC_bru_L37  1 GGTGTGTCTATGAGGTACATTAGCAATGCCTGTGGCTCATGCATAGTTTCTAAAAGAGG 59
JM_Saliva_18    1 GGTGTGTCTATGAGGTACATTAGCAATGCCTGTGGCTCATGCATAGTTTCTAAAAGAGG 59
JM_NKTLY_96.1   1 GGTGTGTCTATGAGGTACATTAGCAATGCCTGTGGCTCATGCATAGTTTCTAAAAGAGG 59
JM_NKTLY_98.1   1 GGTGTGTCTATGAGGTACATTAGCAATGCCTGTGGCTCATGCATAGTTTCTAAAAGAGG 59
RK_LCL_L2       1 GGTGTGTCTATGAGGTACATTAGCAATGCCTGTGGCTCATGCATAGTTTCTAAAAGAGG 59
RK_LCL_L5       1 GGTGTGTCTATGAGGTACATTAGCAATGCCTGTGGCTCATGCATAGTTTCTAAAAGAGG 59
RK_LCL_L12      1 GGTGTGTCTATGAGGTACATTAGCAATGCCTGTGGCTCATGCATAGTTTCTAAAAGAGG 59
RK_LCL_L24      1 GGTGTGTCTATGAGGTACATTAGCAATGCCTGTGGCTCATGCATAGTTTCTAAAAGAGG 59
RK_LCL_H12      1 GGTGTGTCTATGAGGTACATTAGCAATGCCTGTGGCTCATGCATAGTTTCTAAAAGAGG 59
RK_LCL_H16      1 GGTGTGTCTATGAGGTACATTAGCAATGCCTGTGGCTCATGCATAGTTTCTAAAAGAGG 59
RK_LCL_H35      1 GGTGTGTCTATGAGGTACATTAGCAATGCCTGTGGCTCATGCATAGTTTCTAAAAGAGG 59
JM_LCL_IK       1 GGTGTGTCTATGAGGTACATTAGCAATGCCTGTGGCTCATGCATAGTTTCTAAAAGAGG 59
JM_Saliva_33    1 GGTGTGTCTATGAGGTACATTAGCAATGCCTGTGGCTCATGCATAGTTTCTAAAAGAGG 59
GK_BL60         1 GGTGTGTCTATGAGGTACATTAGCAATGCCTGTGGCTCATGCATAGTTTCTAAAAGAGG 59
GK_RUDU         1 GGTGTGTCTATGAGGTACATTAGCAATGCCTGTGGCTCATGCATAGTTTCTAAAAGAGG 59
GK_BL67         1 GGTGTGTCTATGAGGTACATTAGCAATGCCTGTGGCTCATGCATAGTTTCTAAAAGAGG 59
GK_Farage       1 GGTGTGTCTATGAGGTACATTAGCAATGCCTGTGGCTCATGCATAGTTTCTAAAAGAGG 59
GK_K001         1 GGTGTGTCTATGAGGTACATTAGCAATGCCTGTGGCTCATGCATAGTTTCTAAAAGAGG 59
GK_LY65         1 GGTGTGTCTATGAGGTACATTAGCAATGCCTGTGGCTCATGCATAGTTTCTAAAAGAGG 59
GK_BL18         1 GGTGTGTCTATGAGGTACATTAGCAATGCCTGTGGCTCATGCATAGTTTCTAAAAGAGG 59
IMS_Saliva_49   1 GGTGTGTCTATGAGGTACATTAGCAATGCCTGTGGCTCATGCATAGTTTCTAAAAGAGG 59
IMS_Saliva_52   1 GGTGTGTCTATGAGGTACATTAGCAATGCCTGTGGCTCATGCATAGTTTCTAAAAGAGG 59
IMS_Saliva_70   1 GGTGTGTCTATGAGGTACATTAGCAATGCCTGTGGCTCATGCATAGTTTCTAAAAGAGG 59
IMS_Saliva_71   1 GGTGTGTCTATGAGGTACATTAGCAATGCCTGTGGCTCATGCATAGTTTCTAAAAGAGG 59
IMS_Saliva_90   1 GGTGTGTCTATGAGGTACATTAGCAATGCCTGTGGCTCATGCATAGTTTCTAAAAGAGG 59
IMS_Saliva_155  1 GGTGTGTCTATGAGGTACATTAGCAATGCCTGTGGCTCATGCATAGTTTCTAAAAGAGG 59
IMS_Saliva_162  1 GGTGTGTCTATGAGGTACATTAGCAATGCCTGTGGCTCATGCATAGTTTCTAAAAGAGG 59
GK_BL72         1 GGTGTGTCTATGAGGTACATTAGCAATGCCTGTGGCTCATGCATAGTTTCTAAAAGAGG 59
IMS_Saliva_193  1 GGTGTGTCTATGAGGTACATTAGCAATGCCTGTGGCTCATGCATAGTTTCTAAAAGAGG 59
IMS_Saliva_216  1 GGTGTGTCTATGAGGTACATTAGCAATGCCTGTGGCTCATGCATAGTTTCTAAAAGAGG 59
IMS_Saliva_220  1 GGTGTGTCTATGAGGTACATTAGCAATGCCTGTGGCTCATGCATAGTTTCTAAAAGAGG 59
IMS_Saliva_231  1 GGTGTGTCTATGAGGTACATTAGCAATGCCTGTGGCTCATGCATAGTTTCTAAAAGAGG 59
IMS_Saliva_250  1 GGTGTGTCTATGAGGTACATTAGCAATGCCTGTGGCTCATGCATAGTTTCTAAAAGAGG 59
AH_Saliva_8192  1 GGTGTGTCTATGAGGTACATTAGCAATGCCTGTGGCTCATGCATAGTTTCTAAAAGAGG 59
AH_Saliva_8471  1 GGTGTGTCTATGAGGTACATTAGCAATGCCTGTGGCTCATGCATAGTTTCTAAAAGAGG 59
AH_Saliva_8489  1 GGTGTGTCTATGAGGTACATTAGCAATGCCTGTGGCTCATGCATAGTTTCTAAAAGAGG 59
AH_Saliva_9077  1 GGTGTGTCTATGAGGTACATTAGCAATGCCTGTGGCTCATGCATAGTTTCTAAAAGAGG 59
DF_Tonsil_T47   1 GGTGTGTCTATGAGGTACATTAGCAATGCCTGTGGCTCATGCATAGTTTCTAAAAGAGG 59
DF_Tonsil_T49   1 GGTGTGTCTATGAGGTACATTAGCAATGCCTGTGGCTCATGCATAGTTTCTAAAAGAGG 59
DF_Tonsil_T156  1 GGTGTGTCTATGAGGTACATTAGCAATGCCTGTGGCTCATGCATAGTTTCTAAAAGAGG 59
GK_LY47         1 GGTGTGTCTATGAGGTACATTAGCAATGCCTGTGGCTCATGCATAGTTTCTAAAAGAGG 59
IMS_Saliva_5    1 GGTGTGTCTATGAGGTACATTAGCAATGCCTGTGGCTCATGCATAGTTTCTAAAAGAGG 59
JC_027          1 GGTGTGTCTATGAGGTACATTAGCAATGCCTGTGGCTCATGCATAGTTTCTAAAAGAGG 59
JC_030-29       1 GGTGTGTCTATGAGGTACATTAGCAATGCCTGTGGCTCATGCATAGTTTCTAAAAGAGG 59
JC_030-18       1 GGTGTGTCTATGAGGTACATTAGCAATGCCTGTGGCTCATGCATAGTTTCTAAAAGAGG 59
JC_037          1 GGTGTGTCTATGAGGTACATTAGCAATGCCTGTGGCTCATGCATAGTTTCTAAAAGAGG 59
JC_039          1 GGTGTGTCTATGAGGTACATTAGCAATGCCTGTGGCTCATGCATAGTTTCTAAAAGAGG 59
JC_040          1 GGTGTGTCTATGAGGTACATTAGCAATGCCTGTGGCTCATGCATAGTTTCTAAAAGAGG 59
JC_VID14        1 GGTGTGTCTATGAGGTACATTAGCAATGCCTGTGGCTCATGCATAGTTTCTAAAAGAGG 59
JC_VID41        1 GGTGTGTCTATGAGGTACATTAGCAATGCCTGTGGCTCATGCATAGTTTCTAAAAGAGG 59
JW_BL17A        1 GGTGTGTCTATGAGGTACATTAGCAATGCCTGTGGCTCATGCATAGTTTCTAAAAGAGG 59
IMS_Saliva_9    1 GGTGTGTCTATGAGGTACATTAGCAATGCCTGTGGCTCATGCATAGTTTCTAAAAGAGG 59
JW_BL121B       1 GGTGTGTCTATGAGGTACATTAGCAATGCCTGTGGCTCATGCATAGTTTCTAAAAGAGG 59
IMS_Saliva_10   1 GGTGTGTCTATGAGGTACATTAGCAATGCCTGTGGCTCATGCATAGTTTCTAAAAGAGG 59
IMS_Saliva_12   1 GGTGTGTCTATGAGGTACATTAGCAATGCCTGTGGCTCATGCATAGTTTCTAAAAGAGG 59
```

```
NC007605.1        1 GGTGTGTCTATGAGGTACATTAGCAATGCCTGTGGCTCATGCATAGTTTCTAAAAGAGG 59
B958              1 GGTGTGTCTATGAGGTACATTAGCAATGCCTGTGGCTCATGCATAGTTTCTAAAAGAGG 59
Sitoko_2655       1 GGTGTGTCTATGAGGTACATTAGCAATGCCTGTGGCTCATGCATAGTTTCTAAAAGAGG 59
LCL_delEber2_26   1 GGTGTGTCTATGAGGTACATTAGCAATGCCTGTGGCTCATGCATAGTTTCTAAAAGAGG 59
X50-7_2137        1 GGTGTGTCTATGAGGTACATTAGCAATGCCTGTGGCTCATGCATAGTTTCTAAAAGAGG 59
sLCL-IS1.04_211   1 GGTGTGTCTATGAGGTACATTAGCAATGCCTGTGGCTCATGCATAGTTTCTAAAAGAGG 59
sLCL-IS1.18_211   1 GGTGTGTCTATGAGGTACATTAGCAATGCCTGTGGCTCATGCATAGTTTCTAAAAGAGG 59
sLCL-IS1.06_210   1 GGTGTGTCTATGAGGTACATTAGCAATGCCTGTGGCTCATGCATAGTTTCTAAAAGAGG 59
sLCL-IS1.03_210   1 GGTGTGTCTATGAGGTACATTAGCAATGCCTGTGGCTCATGCATAGTTTCTAAAAGAGG 59
sLCL-IS1.11_211   1 GGTGTGTCTATGAGGTACATTAGCAATGCCTGTGGCTCATGCATAGTTTCTAAAAGAGG 59
HL11_3284         1 GGTGTGTCTATGAGGTACATTAGCAATGCCTGTGGCTCATGCATAGTTTCTAAAAGAGG 59
sLCL-IS1.10_211   1 GGTGTGTCTATGAGGTACATTAGCAATGCCTGTGGCTCATGCATAGTTTCTAAAAGAGG 59
sLCL-IS1.08_211   1 GGTGTGTCTATGAGGTACATTAGCAATGCCTGTGGCTCATGCATAGTTTCTAAAAGAGG 59
sLCL-IS1.01_210   1 GGTGTGTCTATGAGGTACATTAGCAATGCCTGTGGCTCATGCATAGTTTCTAAAAGAGG 59
HL02_3275         1 GGTGTGTCTATGAGGTACATTAGCAATGCCTGTGGCTCATGCATAGTTTCTAAAAGAGG 59
L591_3258         1 GGTGTGTCTATGAGGTACATTAGCAATGCCTGTGGCTCATGCATAGTTTCTAAAAGAGG 59
HL08_3281         1 GGTGTGTCTATGAGGTACATTAGCAATGCCTGTGGCTCATGCATAGTTTCTAAAAGAGG 59
pLCL-TRL595_211   1 GGTGTGTCTATGAGGTACATTAGCAATGCCTGTGGCTCATGCATAGTTTCTAAAAGAGG 59
sLCL-1.13_3240    1 GGTGTGTCTATGAGGTACATTAGCAATGCCTGTGGCTCATGCATAGTTTCTAAAAGAGG 59
sLCL-1.24_3250    1 GGTGTGTCTATGAGGTACATTAGCAATGCCTGTGGCTCATGCATAGTTTCTAAAAGAGG 59
sLCL-1.05_3232    1 GGTGTGTCTATGAGGTACATTAGCAATGCCTGTGGCTCATGCATAGTTTCTAAAAGAGG 59
sLCL-1.09_3236    1 GGTGTGTCTATGAGGTACATTAGCAATGCCTGTGGCTCATGCATAGTTTCTAAAAGAGG 59
sLCL-1.17_3244    1 GGTGTGTCTATGAGGTACATTAGCAATGCCTGTGGCTCATGCATAGTTTCTAAAAGAGG 59
sLCL-IS1.12_212   1 GGTGTGTCTATGAGGTACATTAGCAATGCCTGTGGCTCATGCATAGTTTCTAAAAGAGG 59
sLCL-IS1.19_212   1 GGTGTGTCTATGAGGTACATTAGCAATGCCTGTGGCTCATGCATAGTTTCTAAAAGAGG 59
sLCL-IS1.14_212   1 GGTGTGTCTATGAGGTACATTAGCAATGCCTGTGGCTCATGCATAGTTTCTAAAAGAGG 59
sLCL-IS1.07_211   1 GGTGTGTCTATGAGGTACATTAGCAATGCCTGTGGCTCATGCATAGTTTCTAAAAGAGG 59
sLCL-IM1.17_212   1 GGTGTGTCTATGAGGTACATTAGCAATGCCTGTGGCTCATGCATAGTTTCTAAAAGAGG 59
sLCL-1.12_3239    1 GGTGTGTCTATGAGGTACATTAGCAATGCCTGTGGCTCATGCATAGTTTCTAAAAGAGG 59
sLCL-IS1.20_212   1 GGTGTGTCTATGAGGTACATTAGCAATGCCTGTGGCTCATGCATAGTTTCTAAAAGAGG 59
sLCL-1.04_3231    1 GGTGTGTCTATGAGGTACATTAGCAATGCCTGTGGCTCATGCATAGTTTCTAAAAGAGG 59
Daudi_2145        1 GGTGTGTCTATGAGGTACATTAGCAATGCCTGTGGCTCATGCATAGTTTCTAAAAGAGG 59
M-ABA_2139        1 GGTGTGTCTATGAGGTACATTAGCAATGCCTGTGGCTCATGCATAGTTTCTAAAAGAGG 59
sLCL-BL1.03_323   1 GGTGTGTCTATGAGGTACATTAGCAATGCCTGTGGCTCATGCATAGTTTCTAAAAGAGG 59
sLCL-1.06_3233    1 GGTGTGTCTATGAGGTACATTAGCAATGCCTGTGGCTCATGCATAGTTTCTAAAAGAGG 59
sLCL-IM1.09_212   1 GGTGTGTCTATGAGGTACATTAGCAATGCCTGTGGCTCATGCATAGTTTCTAAAAGAGG 59
HL04_3277         1 GGTGTGTCTATGAGGTACATTAGCAATGCCTGTGGCTCATGCATAGTTTCTAAAAGAGG 59
sLCL-1.11_3238    1 GGTGTGTCTATGAGGTACATTAGCAATGCCTGTGGCTCATGCATAGTTTCTAAAAGAGG 59
sLCL-1.07_3234    1 GGTGTGTCTATGAGGTACATTAGCAATGCCTGTGGCTCATGCATAGTTTCTAAAAGAGG 59
sLCL-1.10_3237    1 GGTGTGTCTATGAGGTACATTAGCAATGCCTGTGGCTCATGCATAGTTTCTAAAAGAGG 59
sLCL-BL1.20_324   1 GGTGTGTCTATGAGGTACATTAGCAATGCCTGTGGCTCATGCATAGTTTCTAAAAGAGG 59
sLCL-1.08_3235    1 GGTGTGTCTATGAGGTACATTAGCAATGCCTGTGGCTCATGCATAGTTTCTAAAAGAGG 59
sLCL-1.02_3229    1 GGTGTGTCTATGAGGTACATTAGCAATGCCTGTGGCTCATGCATAGTTTCTAAAAGAGG 59
BL37_2650         1 GGTGTGTCTATGAGGTACATTAGCAATGCCTGTGGCTCATGCATAGTTTCTAAAAGAGG 59
HL01_3274         1 GGTGTGTCTATGAGGTACATTAGCAATGCCTGTGGCTCATGCATAGTTTCTAAAAGAGG 59
HL09_3282         1 GGTGTGTCTATGAGGTACATTAGCAATGCCTGTGGCTCATGCATAGTTTCTAAAAGAGG 59
sLCL-IM1.02_210   1 GGTGTGTCTATGAGGTACATTAGCAATGCCTGTGGCTCATGCATAGTTTCTAAAAGAGG 59
sLCL-IM1.05_210   1 GGTGTGTCTATGAGGTACATTAGCAATGCCTGTGGCTCATGCATAGTTTCTAAAAGAGG 59
HL05_3278         1 GGTGTGTCTATGAGGTACATTAGCAATGCCTGTGGCTCATGCATAGTTTCTAAAAGAGG 59
YCCEL1_3256       1 GGTGTGTCTATGAGGTACATTAGCAATGCCTGTGGCTCATGCATAGTTTCTAAAAGAGG 59
sLCL-IS1.13_210   1 GGTGTGTCTATGAGGTACATTAGCAATGCCTGTGGCTCATGCATAGTTTCTAAAAGAGG 59
sLCL-IM1.16_211   1 GGTGTGTCTATGAGGTACATTAGCAATGCCTGTGGCTCATGCATAGTTTCTAAAAGAGG 59
Raji              1 GGTGTGTCTATGAGGTACATTAGCAATGCCTGTGGCTCATGCATAGTTTCTAAAAGAGG 59
Mutu              1 GGTGTGTCTATGAGGTACATTAGCAATGCCTGTGGCTCATGCATAGTTTCTAAAAGAGG 59
HKN14_3252        1 GGTGTGTCTATGAGGTACATTAGCAATGCCTGTGGCTCATGCATAGTTTCTAAAAGAGG 59
D3201.2_3259      1 GGTGTGTCTATGAGGTACATTAGCAATGCCTGTGGCTCATGCATAGTTTCTAAAAGAGG 59
sLCL-1.19_3246    1 GGTGTGTCTATGAGGTACATTAGCAATGCCTGTGGCTCATGCATAGTTTCTAAAAGAGG 59
K4123             1 GGTGTGTCTATGAGGTACATTAGCAATGCCTGTGGCTCATGCATAGTTTCTAAAAGAGG 59
K4413             1 GGTGTGTCTATGAGGTACATTAGCAATGCCTGTGGCTCATGCATAGTTTCTAAAAGAGG 59
JM_NPC_biop_27    1 GGTGTGTCTATGAGGTACATTAGCAATGCCTGTGGCTCATGCATAGTTTCTAAAAGAGG 59
Rael AJ422219     1 GGTGTGTCTATGAGGTACATTAGCAATGCCTGTGGCTCATGCATAGTTTCTAAAAGAGG 60
                    ********* ***** ********************************** ********

CCC
                    ********* ***** ********************************** ********
JM_NPC_biop_51    1 GGTGTGTCTCTGAGGCACATTAGCAATGCCTGTGGCTCATGCATAGTTTCCAAAAGAGG 59
JM_NPC_biop_162   1 GGTGTGTCTCTGAGGCACATTAGCAATGCCTGTGGCTCATGCATAGTTTCCAAAAGAGG 59
JM_NPC_biop_238   1 GGTGTGTCTCTGAGGCACATTAGCAATGCCTGTGGCTCATGCATAGTTTCCAAAAGAGG 59
JM_NPC_bru_L3     1 GGTGTGTCTCTGAGGCACATTAGCAATGCCTGTGGCTCATGCATAGTTTCCAAAAGAGG 59
JM_NPC_bru_L29    1 GGTGTGTCTCTGAGGCACATTAGCAATGCCTGTGGCTCATGCATAGTTTCCAAAAGAGG 59
JM_NPC_bru_L41    1 GGTGTGTCTCTGAGGCACATTAGCAATGCCTGTGGCTCATGCATAGTTTCCAAAAGAGG 59
JM_NPC_bru_L42    1 GGTGTGTCTCTGAGGCACATTAGCAATGCCTGTGGCTCATGCATAGTTTCCAAAAGAGG 59
```

```
JM_NPC_bru_L47    1 GGTGTGTCTCTGAGGCACATTAGCAATGCCTGTGGCTCATGCATAGTTTCCAAAAGAGG 59
JM_NKTLY_97.1     1 GGTGTGTCTCTGAGGCACATTAGCAATGCCTGTGGCTCATGCATAGTTTCCAAAAGAGG 59
JM_NKTLY_218.1    1 GGTGTGTCTCTGAGGCACATTAGCAATGCCTGTGGCTCATGCATAGTTTCCAAAAGAGG 59
JM_LCL_MU         1 GGTGTGTCTCTGAGGCACATTAGCAATGCCTGTGGCTCATGCATAGTTTCCAAAAGAGG 59
JM_LCL_SU         1 GGTGTGTCTCTGAGGCACATTAGCAATGCCTGTGGCTCATGCATAGTTTCCAAAAGAGG 59
JM_LCL_IN         1 GGTGTGTCTCTGAGGCACATTAGCAATGCCTGTGGCTCATGCATAGTTTCCAAAAGAGG 59
GK_Akuba          1 GGTGTGTCTCTGAGGCACATTAGCAATGCCTGTGGCTCATGCATAGTTTCCAAAAGAGG 59
GK_BL42           1 GGTGTGTCTCTGAGGCACATTAGCAATGCCTGTGGCTCATGCATAGTTTCCAAAAGAGG 59
GK_BL44           1 GGTGTGTCTCTGAGGCACATTAGCAATGCCTGTGGCTCATGCATAGTTTCCAAAAGAGG 59
JM_NPC_bru_38     1 GGTGTGTCTCTGAGGCACATTAGCAATGCCTGTGGCTCATGCATAGTTTCCAAAAGAGG 59
JM_NPC_bru_51     1 GGTGTGTCTCTGAGGCACATTAGCAATGCCTGTGGCTCATGCATAGTTTCCAAAAGAGG 59
JM_NPC_bru_178    1 GGTGTGTCTCTGAGGCACATTAGCAATGCCTGTGGCTCATGCATAGTTTCCAAAAGAGG 59
JM_NPC_bru_238    1 GGTGTGTCTCTGAGGCACATTAGCAATGCCTGTGGCTCATGCATAGTTTCCAAAAGAGG 59
JM_NPC_bru_377    1 GGTGTGTCTCTGAGGCACATTAGCAATGCCTGTGGCTCATGCATAGTTTCCAAAAGAGG 59
IMS_Saliva_120    1 GGTGTGTCTCTGAGGCACATTAGCAATGCCTGTGGCTCATGCATAGTTTCCAAAAGAGG 59
IMS_Saliva_170    1 GGTGTGTCTCTGAGGCACATTAGCAATGCCTGTGGCTCATGCATAGTTTCCAAAAGAGG 59
IMS_Saliva_177    1 GGTGTGTCTCTGAGGCACATTAGCAATGCCTGTGGCTCATGCATAGTTTCCAAAAGAGG 59
IMS_Saliva_187    1 GGTGTGTCTCTGAGGCACATTAGCAATGCCTGTGGCTCATGCATAGTTTCCAAAAGAGG 59
IMS_Saliva_204    1 GGTGTGTCTCTGAGGCACATTAGCAATGCCTGTGGCTCATGCATAGTTTCCAAAAGAGG 59
IMS_Saliva_243    1 GGTGTGTCTCTGAGGCACATTAGCAATGCCTGTGGCTCATGCATAGTTTCCAAAAGAGG 59
IMS_Saliva_248    1 GGTGTGTCTCTGAGGCACATTAGCAATGCCTGTGGCTCATGCATAGTTTCCAAAAGAGG 59
M81_gDNA          1 GGTGTGTCTCTGAGGCACATTAGCAATGCCTGTGGCTCATGCATAGTTTCCAAAAGAGG 59
JC_002            1 GGTGTGTCTCTGAGGCACATTAGCAATGCCTGTGGCTCATGCATAGTTTCCAAAAGAGG 59
JC_023            1 GGTGTGTCTCTGAGGCACATTAGCAATGCCTGTGGCTCATGCATAGTTTCCAAAAGAGG 59
IMS_Saliva_6      1 GGTGTGTCTCTGAGGCACATTAGCAATGCCTGTGGCTCATGCATAGTTTCCAAAAGAGG 59
JC_V005           1 GGTGTGTCTCTGAGGCACATTAGCAATGCCTGTGGCTCATGCATAGTTTCCAAAAGAGG 59
JC_V006           1 GGTGTGTCTCTGAGGCACATTAGCAATGCCTGTGGCTCATGCATAGTTTCCAAAAGAGG 59
JW_BL43B          1 GGTGTGTCTCTGAGGCACATTAGCAATGCCTGTGGCTCATGCATAGTTTCCAAAAGAGG 59
IMS_Saliva_31     1 GGTGTGTCTCTGAGGCACATTAGCAATGCCTGTGGCTCATGCATAGTTTCCAAAAGAGG 59
pLCL-TRL1-post_   1 GGTGTGTCTCTGAGGCACATTAGCAATGCCTGTGGCTCATGCATAGTTTCCAAAAGAGG 59
pLCL-TRL1-pre_2   1 GGTGTGTCTCTGAGGCACATTAGCAATGCCTGTGGCTCATGCATAGTTTCCAAAAGAGG 59
Mak_1_2129        1 GGTGTGTCTCTGAGGCACATTAGCAATGCCTGTGGCTCATGCATAGTTTCCAAAAGAGG 59
Akata_2000_2128   1 GGTGTGTCTCTGAGGCACATTAGCAATGCCTGTGGCTCATGCATAGTTTCCAAAAGAGG 59
Makau_2649        1 GGTGTGTCTCTGAGGCACATTAGCAATGCCTGTGGCTCATGCATAGTTTCCAAAAGAGG 59
sLCL-IS1.15_212   1 GGTGTGTCTCTGAGGCACATTAGCAATGCCTGTGGCTCATGCATAGTTTCCAAAAGAGG 59
C666.1imp_2648    1 GGTGTGTCTCTGAGGCACATTAGCAATGCCTGTGGCTCATGCATAGTTTCCAAAAGAGG 59
HKN15_3253        1 GGTGTGTCTCTGAGGCACATTAGCAATGCCTGTGGCTCATGCATAGTTTCCAAAAGAGG 59
HKN19_3254        1 GGTGTGTCTCTGAGGCACATTAGCAATGCCTGTGGCTCATGCATAGTTTCCAAAAGAGG 59
C666              1 GGTGTGTCTCTGAGGCACATTAGCAATGCCTGTGGCTCATGCATAGTTTCCAAAAGAGG 59
GD2               1 GGTGTGTCTCTGAGGCACATTAGCAATGCCTGTGGCTCATGCATAGTTTCCAAAAGAGG 59
sLCL-1.18_3245    1 GGTGTGTCTCTGAGGCACATTAGCAATGCCTGTGGCTCATGCATAGTTTCCAAAAGAGG 59
GD1               1 GGTGTGTCTCTGAGGCACATTAGCAATGCCTGTGGCTCATGCATAGTTTCCAAAAGAGG 59
Akata             1 GGTGTGTCTCTGAGGCACATTAGCAATGCCTGTGGCTCATGCATAGTTTCCAAAAGAGG 59
HKNPC1            1 GGTGTGTCTCTGAGGCACATTAGCAATGCCTGTGGCTCATGCATAGTTTCCAAAAGAGG 59
BL36_2652         1 GGTGTGTCTCTGAGGCACATTAGCAATGCCTGTGGCTCATGCATAGTTTCCAAAAGAGG 59
M81               1 GGTGTGTCTCTGAGGCACATTAGCAATGCCTGTGGCTCATGCATAGTTTCCAAAAGAGG 59
Namalwa_2131      1 GGTGTGTCTCTGAGGCACATTAGCAATGCCTGTGGCTCATGCATAGTTTCCAAAAGAGG 59

Type 1/2
GK_BL36           1 GGTGTGTCTCTGAGGCACATTAGCAATGCCTGTGGCTCATGCATAGTTTCCAAAAGAGG 59

Type 2

AG876             1 GGTGTGTCTCTGAGGCACATTAGCAATGCCTGTGGCTCATGCATAGTTTCCAAAAGAGG 59
IMS_Saliva_81     1 GGTGTGTCTCTGAGGCACATTAGCAATGCCTGTGGCTCATGCATAGTTTCCAAAAGAGG 59
JM_NPC_bru_L4     1 GGTGTGTCTCTGAGGCACATTAGCAATGCCTGTGGCTCATGCATAGTTTCCAAAAGAGG 59
JM_NPC_bru_L7     1 GGTGTGTCTCTGAGGCACATTAGCAATGCCTGTGGCTCATGCATAGTTTCCAAAAGAGG 59
RK_LCL_L3         1 GGTGTGTCTCTGAGGCACATTAGCAATGCCTGTGGCTCATGCATAGTTTCCAAAAGAGG 59
RK_LCL_L4         1 GGTGTGTCTCTGAGGCACATTAGCAATGCCTGTGGCTCATGCATAGTTTCCAAAAGAGG 59
JM_Saliva_20      1 GGTGTGTCTCTGAGGCACATTAGCAATGCCTGTGGCTCATGCATAGTTTCCAAAAGAGG 59
RK_LCL_L19        1 GGTGTGTCTCTGAGGCACATTAGCAATGCCTGTGGCTCATGCATAGTTTCCAAAAGAGG 59
GK_PUT            1 GGTGTGTCTCTGAGGCACATTAGCAATGCCTGTGGCTCATGCATAGTTTCCAAAAGAGG 59
GK_LY91           1 GGTGTGTCTCTGAGGCACATTAGCAATGCCTGTGGCTCATGCATAGTTTCCAAAAGAGG 59
GK_BL16           1 GGTGTGTCTCTGAGGCACATTAGCAATGCCTGTGGCTCATGCATAGTTTCCAAAAGAGG 59
AH_Saliva_9316    1 GGTGTGTCTCTGAGGCACATTAGCAATGCCTGTGGCTCATGCATAGTTTCCAAAAGAGG 59
AFB1_2645         1 GGTGTGTCTCTGAGGCACATTAGCAATGCCTGTGGCTCATGCATAGTTTCCAAAAGAGG 59
Cheptages_2143    1 GGTGTGTCTCTGAGGCACATTAGCAATGCCTGTGGCTCATGCATAGTTTCCAAAAGAGG 59
sLCL-2.21_3248    1 GGTGTGTCTCTGAGGCACATTAGCAATGCCTGTGGCTCATGCATAGTTTCCAAAAGAGG 59
sLCL-2.14_3241    1 GGTGTGTCTCTGAGGCACATTAGCAATGCCTGTGGCTCATGCATAGTTTCCAAAAGAGG 59
sLCL-2.15_3242    1 GGTGTGTCTCTGAGGCACATTAGCAATGCCTGTGGCTCATGCATAGTTTCCAAAAGAGG 59
sLCL-2.16_3243    1 GGTGTGTCTCTGAGGCACATTAGCAATGCCTGTGGCTCATGCATAGTTTCCAAAAGAGG 59
sLCL-2.22_3249    1 GGTGTGTCTCTGAGGCACATTAGCAATGCCTGTGGCTCATGCATAGTTTCCAAAAGAGG 59
```

```
sLCL-IS2.01_212  1 GGTGTGTCTCTGAGGCACATTAGCAATGCCTGTGGCTCATGCATAGTTTCCAAAAGAGG 59
P3HR1_c16_2134   1 GGTGTGTCTCTGAGGCACATTAGCAATGCCTGTGGCTCATGCATAGTTTCCAAAAGAGG 59
Jijoye_2135      1 GGTGTGTCTCTGAGGCACATTAGCAATGCCTGTGGCTCATGCATAGTTTCCAAAAGAGG 59
Wewak_2          1 GGTGTGTCTCTGAGGCACATTAGCAATGCCTGTGGCTCATGCATAGTTTCCAAAAGAGG 59
                   ********* ***** ********************************* ********
```

Figure S6 Phylogenetic tree of BART cluster 2 sequences summarised in Fig 5
Method: Neighbor Joining; Best Tree; tie breaking = Systematic
Distance: Uncorrected ("p"). Gaps distributed proportionally

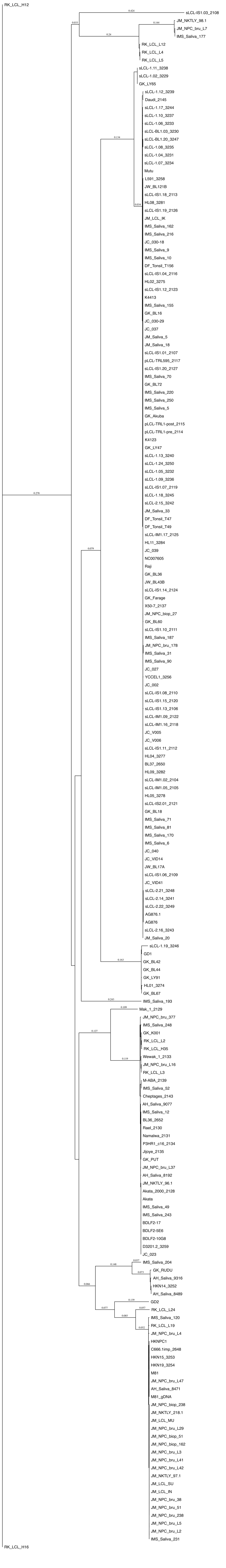

Supplement: Supplemental material [file JVI.00375-17_zjv999182767s1.pdf]
